# Obstructive sleep apnea and attention deficits: A systematic review of magnetic resonance imaging biomarkers and neuropsychological assessments

**DOI:** 10.1002/brb3.3262

**Published:** 2023-09-24

**Authors:** Sadegh Ghaderi, Sana Mohammadi, Mahdi Mohammadi

**Affiliations:** ^1^ Department of Neuroscience and Addiction Studies School of Advanced Technologies in Medicine Tehran University of Medical Sciences Tehran Iran; ^2^ Department of Medical Sciences School of Medicine Iran University of Medical Sciences Tehran Iran; ^3^ Department of Medical Physics and Biomedical Engineering, School of Medicine Tehran University of Medical Sciences Tehran Iran

**Keywords:** attention deficit, MRI, MRI biomarkers, neuropsychological tests, OSA

## Abstract

**Background and objective:**

Obstructive sleep apnea (OSA) is a common sleep disorder that causes intermittent hypoxia and sleep fragmentation, leading to attention impairment and other cognitive deficits. Magnetic resonance imaging (MRI) is a powerful modality that can reveal the structural and functional brain alterations associated with attention impairment in OSA patients. The objective of this systematic review is to identify and synthesize the evidence on MRI biomarkers and neuropsychological assessments of attention deficits in OSA patients.

**Methods:**

We searched the Scopus and PubMed databases for studies that used MRI to measure biomarkers related to attention alteration in OSA patients and reported qualitative and quantitative data on the association between MRI biomarkers and attention outcomes. We also included studies that found an association between neuropsychological assessments and MRI findings in OSA patients with attention deficits.

**Results:**

We included 19 studies that met our inclusion criteria and extracted the relevant data from each study. We categorized the studies into three groups based on the MRI modality and the cognitive domain they used: structural and diffusion tensor imaging MRI findings, functional, perfusion, and metabolic MRI findings, and neuropsychological assessment findings.

**Conclusions:**

We found that OSA is associated with structural, functional, and metabolic brain alterations in multiple regions and networks that are involved in attention processing. Treatment with continuous positive airway pressure can partially reverse some of the brain changes and improve cognitive function in some domains and in some studies. This review suggests that MRI techniques and neuropsychological assessments can be useful tools for monitoring the progression and response to treatment of OSA patients.

## Graphical abstract

This systematic review analyzes MRI biomarkers and neuropsychological assessments related to attention deficits in obstructive sleep apnea patients. The findings suggest obstructive sleep apnea is associated with structural and functional brain changes in regions involved in attention processing, which correlate with disease severity and cognitive impairment.

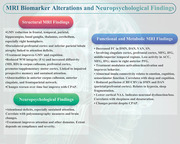

## 1 INTRODUCTION

Obstructive sleep apnea (OSA) is a prevalent sleep disorder characterized by recurrent episodes of upper airway collapse during sleep, leading to intermittent hypoxia and sleep fragmentation (Bhuniya et al., 2022; Carvalho et al., 2023; Ji et al., 2022; Lal et al., 2021; Martins & Conde, 2021; Slowik et al., 2023). It affects millions of people worldwide (Benjafield et al., 2019; Lyons et al., 2020). OSA affects 33% of men and 16% of women in the adult population and is associated with an increased risk of cardiovascular, metabolic, and neurocognitive complications (Abbasi et al., 2021; Barletta et al., 2019; Lal et al., 2022; Liu et al., 2020; Wang et al., 2022). Among the neurocognitive consequences of OSA, attention impairment is one of the most frequently reported and disabling symptoms, impacting the quality of life and daily functioning of OSA patients (Angelelli et al., 2020; Bilyukov et al., 2018; Bucks et al., 2013; Krysta et al., 2017). Attention is a complex cognitive function that encompasses multiple processes and involves various brain regions. Attention impairment in OSA patients can affect different types of attention (Angelelli et al., 2020; Krysta et al., 2017; Simões et al., 2018).

The standard therapy for OSA is continuous positive airway pressure (CPAP), which delivers pressurized air through a mask to keep the airway open during sleep (Cao et al., 2017; Spicuzza et al., 2015). CPAP can improve the quality of sleep, reduce daytime symptoms, and lower the risk of complications associated with OSA (Donovan et al., 2015; Spicuzza et al., 2015). The underlying mechanisms of attention impairment in OSA remain unclear (Ji et al., 2021), but several factors can contribute, such as excessive daytime sleepiness (EDS) (Steiropoulos et al., 2019), nocturnal hypoxia (Liu et al., 2020), oxidative stress (Angelelli et al., 2020), inflammation (Liu et al., 2020), and vascular damage (Kujovic et al., 2023). Additionally, structural and functional alterations in brain regions involved in attention processes have been observed in OSA patients using magnetic resonance imaging (MRI) techniques (Shin et al., 2013; Tahmasian et al., 2016; Volner et al., 2022).

MRI is a powerful, noninvasive modality that offers information on the anatomy, physiology, metabolism, and connectivity of the brain (Clayden, 2013; Ghaderi et al., 2023; Mohammadi et al., 2023; Yousaf et al., 2018). MRI measures biomarkers reflecting various aspects of brain structure and function (Lin et al., 2011; Steffener et al., 2012), such as gray matter volume (GMV) (Liu, 2015), white matter (WM) integrity (Mohtasib et al., 2022), cerebral blood flow (CBF) (Song et al., 2022), glucose metabolism (Xie et al., 2023), oxygen extraction fraction (Lundberg et al., 2022), and functional connectivity (FC) (Spina et al., 2019).

Advanced MRI techniques, such as volumetric analysis, diffusion tensor imaging (DTI), perfusion‐weighted imaging (PWI), susceptibility‐weighted imaging, functional MRI (fMRI), and magnetic resonance spectroscopy (MRS), can reveal the microstructural, functional, and metabolic alterations in brain regions involved in attention processes, such as the prefrontal cortex, the anterior cingulate cortex (ACC), the parietal cortex, and the thalamus, in OSA patients (Holdsworth & Bammer, 2008; Jiang & Lu, 2022; Parrino et al., 2022; Volner et al., 2022).

These regions are part of a network that supports attention control and allocation (Ayalon et al., 2009; Canessa et al., 2011). Furthermore, advanced MRI techniques can also show the potential recovery of brain structure and function after CPAP treatment (Chen et al., 2017; Xiong et al., 2017). In total, the objective of this systematic review is to identify and synthesize the evidence on MRI biomarkers and neuropsychological assessments of attention deficits in OSA patients.

## 2 METHODS

### 2.1 Search strategy

**FIGURE 1:**
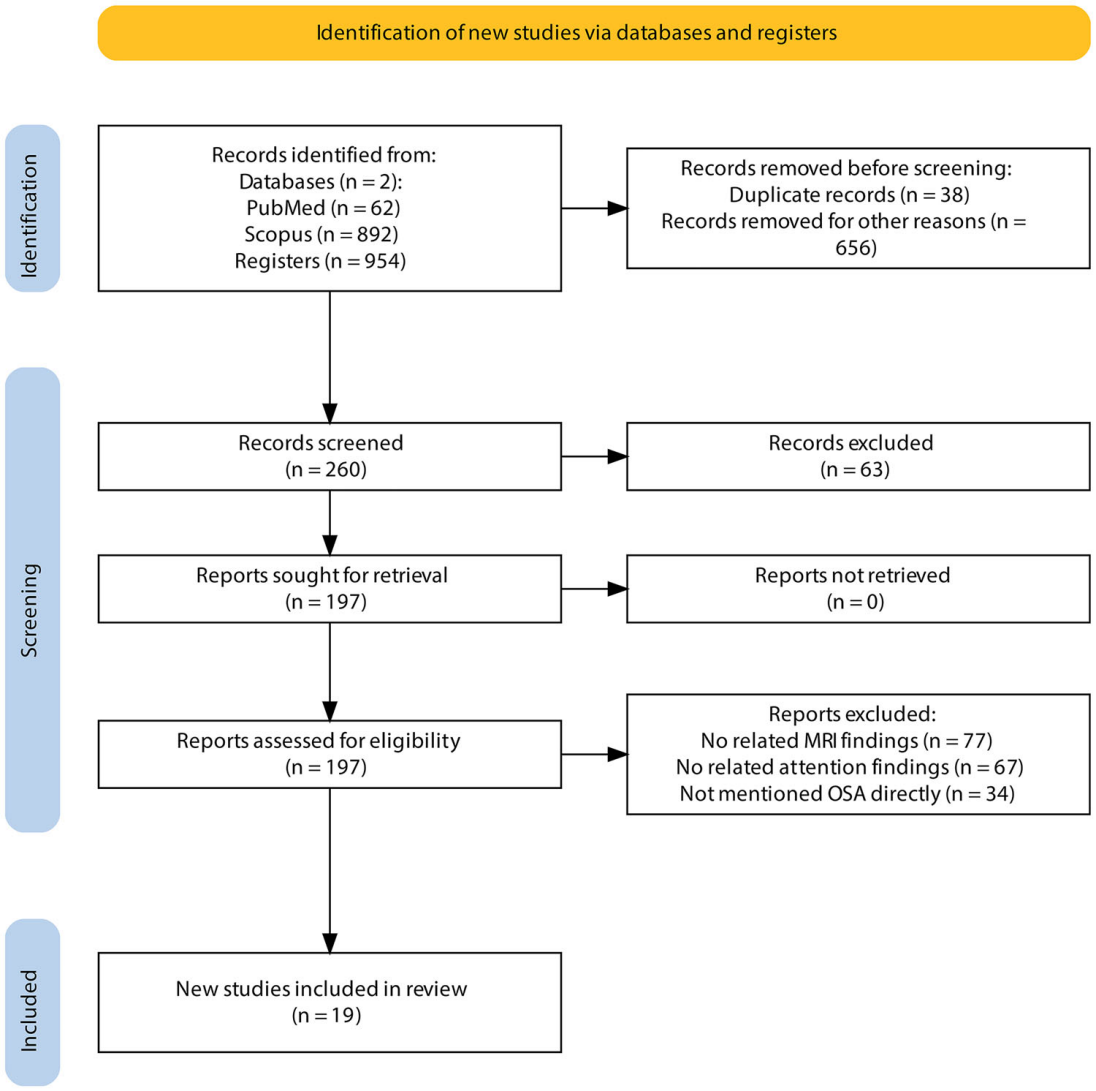
PRISMA flow diagram of the study selection process.

The systematic review selection process is summarized in the reporting in systematic reviews and meta‐analyses(PRISMA) flow diagram guidelines (Figure 1) (Page et al., 2021). We conducted a comprehensive search of the Scopus and PubMed databases in all field formats to identify relevant studies. We used a combination of free‐text and controlled vocabulary terms related to OSA, MRI, and attention impairment, disorder, loss, and deficit. The search was limited to studies published in English and included articles published up to June 2023.

### 2.2 Study selection

All reviewers independently assessed the eligibility of the retrieved studies based on the inclusion and exclusion criteria. Studies that met the following criteria were included: (1) Participants were adults diagnosed with OSA according to established criteria (ages more than 16‐year old and definitive diagnosis with PSG device); (2) MRI was used to measure biomarkers related to attention alteration in OSA patients; (3) the studies reported qualitative and quantitative data on the association between neuropsychological assessments and MRI findings in OSA patients with attention deficits; (4) the studies were published in English. We excluded studies that (1) used other imaging modalities besides MRI; (2) measured biomarkers unrelated to attention impairment; (3) did not report qualitative and quantitative data on the association between MRI biomarkers and attention outcomes; or (4) were reviews, meta‐analyses, case reports, editorials, letters, or conference abstracts.

### 2.3 Data extraction and quality assessment

One reviewer (M.M.) independently extracted the following data from each included study using a standardized form: study characteristics (first author and year, design, sample size, inclusion, and exclusion criteria), participant characteristics (age, sex, body mass index, apnea‐hypopnea index (AHI), and neuropsychological assessments), MRI characteristics (field strength, coil, and imaging techniques), and main findings. Any discrepancies were resolved through discussion with other reviewers. Two reviewers (S.M. and S.Gh.) independently assessed the quality of the included studies using the Cochrane Risk of Bias tool for randomized controlled trials (Higgins et al., 2011) and the Newcastle–Ottawa Scale for observational studies (Lo et al., 2014).

### 2.4 Analysis of global population

In addition to the literature review, we conducted an analysis of the global representation and population diversity of the included studies on OSA, MRI measures, and neuropsychological assessments. The first author's country of origin was recorded for each study. Frequencies were calculated to quantify the contribution of different countries and regions to the overall literature. This allowed the assessment of geographical patterns in study locations and the identification of over‐ or underrepresentation of particular global patient populations.

## 3 RESULTS

### 3.1 Overview of the included studies

The search strategy yielded 954 studies from the electronic databases. Of these, 694 records were excluded for various reasons (see Figure 1 for details). After the first screening, 260 studies were screened by title and abstract, and 63 of them were excluded. After an assessment of eligibility, 177 records were excluded because they did not meet the eligibility criteria. Finally, 19 studies were included in the systematic review. The studies were published between 2007 and 2022. The participant characteristics and study methodologies are summarized in Table 1.

**TABLE 1:** Magnetic resonance imaging (MRI) and neuropsychological findings of attention deficits in obstructive sleep apnea (OSA) patients.

Study	Patients/Controls	Mean BMI/AHI		Neuropsychological and/or clinical assessments	Imaging		MRI and neuropsychological findings
		Patients	Controls		Device	Technique (s)	
Tonon et al. (2007)	14/10	32.3/NA	25/NA	VPSG and neuropsychological tests before and after 6 months of CPAP (alongside MSLT measures)	1.5 T and quadrature birdcage head coil	MRS	● ↓ Cortical NAA concentration at baseline than HCs ● Cortical NAA concentration ↔ minimum SpO_2_ and shorter sleep latency on MSLT score ● CPAP therapy for 6 months does not change cortical NAA, Cr, and Cho concentrations, even with improved EDS, arousals, and O_2_ saturation
Yaouhi et al. (2009)	16/14	NA/38.3	NA/5.7	PSG, MWT, and attention/vigilance NP tests, including bimodal vigilance, alertness, go/no‐go, visual scanning, forward digit, and spatial spans	1.5 T	T1‐w and metabolic measure	● ↓ GM in multiple brain regions, mostly in right hemisphere ● ↓ Brain metabolism in right‐lateralized regions, including precuneus, cingulate gyrus, parietooccipital cortex, and prefrontal cortex ● Despite ↓ GM density and metabolic levels, attention performance remained normal on neuropsychological testing
Canessa et al. (2011)	17/15	31.2/55.8	26.1/1.6	PSG at baseline and 3 months post‐CPAP with NP tests for attention, EES, BDI, and SF‐36	3 T	T1‐w	● Pretreatment brain MRI showed ↓ GMV in left hippocampus, left parietal cortex, and right frontal gyrus ● Posttreatment cognition (attention) ↑ → GMV ↑ in hippocampus and frontal regions ● OSA → attention and structural deficits ← sleep deprivation and hypoxemia
Zhang et al. (2011)	9/9	35.7/28.3	23.1/1.2	Visual DMS task during PSG with geometric shapes as stimuli in match/mismatch conditions	1.5 T	fMRI	● ↓ Frontal activation in ACCs, MFGs, and IFGs, and ↑ right aPFG activation in mismatch tasks ● Oxygen desaturation duration and arousal index ↔ slower reaction times and ↓ frontal activation in ROIs for OSAS patients in mismatch tasks ● Slower reaction times in mismatch tasks → more sustained attention and time needed for mismatch information processing
Torelli et al. (2011)	16/14	31.7/52.5	25.5/NA	Nocturnal cardiorespiratory monitoring with NP evaluations, including Stroop Color/Word Test, and Digit Span backward	3 T	3D T1‐w, 3D T2‐w, and 3D T2‐FLAIR	● ↓ Cortical GM, right hippocampus, and bilateral caudate volumes in moderate‐severe OSA patients than HCs ● ↓ GM volume in bilateral hippocampus and lateral temporal areas in OSA patients
Prilipko et al. (2012)	9 (Active‐CPAP)/8 (Sham‐CPAP)	29.9/45.8	25.5/32.8	PSG and n‐back task performed during the fMRI session	3 T	fMRI	● ↓ Deactivation in temporal regions of DMN, but normal activation in TPN regions, in OSA compared to HCs ● Posttreatment, active CPAP group and sham CPAP group ↓↑ cerebral activation and deactivation ● ↑ Cerebral activation in TPN and ↑ deactivation in anterior midline and medial temporal regions of DMN at 3‐back level, → improved behavioral performance in active CPAP group ● ↓ Deactivation in temporal regions of DMN and ↓ TPN activation, → longer response times at 3‐back in sham CPAP group ● ↓ Vigilance, stimulus detection, and attention shifting in OSA patients
Joo et al. (2013)	38/36	26.1/53.5	25.8/2.8	PSG with attention/working memory tests: Wechsler memory scale‐revised, Rey complex figure test, and Corsi block tapping tests	3 T and 16‐channel head coil	3D T1‐w, 3D T2‐w, and FLAIR	● ↓ Cortical thickness in dorsolateral prefrontal regions and left inferior parietal lobule ● Dorsolateral prefrontal cortex and inferior parietal lobule → attention processing ● Higher arousal indices and longer apnea duration ↔ ↓ cortical thickness of temporal regions and ↓ verbal and visual attention in OSA patients
Castronovo et al. (2014)	15/13	29.9/61.3	26.1/1.6	PSG followed by CPAP treatment for 3 months and 1 year, with NP evaluation, including: MMSE (general cognitive function), trail making test (divided attention), Stroop test (executive functions, inhibition, selective attention), and PASAT (vigilance, executive functions)	3 T and 8‐channel SENSE head coil	DTI	● ↓ Cognition, mood, and sleepiness, ↔ ↓ WM fiber integrity (FA and MD) in multiple brain areas in pretreatment OSA patients ● WM changes after 3 months of CPAP: limited changes in WM ● WM changes after 12 months of CPAP: ↔ in compliant patients ● WM changes after treatment → ↑ attention and executive functioning ● CPAP compliance: ↑ positive outcomes
Rosenzweig et al. (2016)	55/35	34.7/36.6	27.8/NA	PSG followed by 1‐month CPAP treatment, with cognitive function evaluated using: ACE‐R, TMA, TMB, LMT (immediate/delayed with alternate stories), and Wechsler Memory Scale subtests (DST forward/backward, spatial span subtest forward/backward)	1.5 T	3D T1‐w MPRAGE	● CPAP + BSC (1 month) → ↑ right thalamus size (vs. BSC only) ● CPAP + BSC → ↓ ESS, ↑ brainstem, and memory (↔) ● Neuroplasticity ↔ ↑ attention and working memory ● OSA → ↑ EDS and↓ attention
Lin et al. (2016)	21/15	26.2/38.7	23.9/2.4	PSG with NP tests evaluating attention and executive function	3 T and 8‐channel head coil	3D T1‐FLAIR	● Cases versus controls → ↓ anterior cingulate GMV ● After treatment → ↓ GMVs in precuneus, insula, and cerebellum (recovery from edema) ● After treatment → ↑ memory, attention, and executive‐functioning, ↑ hippocampal and frontal GMV
Zhang et al. (2019)	20/24	26.3/49	22.8/NA	PSG with cognitive tests for prospective memory and sustained attention, with sustained attention measured using a computerized version of the continuous performance task‐identical pairs	3 T and 24‐channel head coil	DTI	● OSA versus HCs → ↓ WM integrity in anterior CC ● Prefrontal cortex → ↑ RD ● Premotor and supplementary motor cortex → ↓ FA, ↑ MD and RD ● ↓ Anterior CC integrity ↔ ↓ prospective memory and sustained attention
Chang et al. (2020)	28/–	NA/39.2	NA/NA	PSG with evaluation of general cognitive performance using CDR and CASI, with CDR used to rate performances in six domains, and CASI used to evaluate specific domains including short‐term memory, long‐term memory, orientation, attention/concentration, abstraction, visual construction, language, and list‐generating fluency	3 T	rs‐fMRI	● DMN FC ↔ AHI, ODI, and nadir SaO_2_ (%) ● AHI, ODI, and nadir SaO_2_ (%) ↔ FC in bilateral middle temporal gyri, frontal pole, and hippocampus ● FC in these areas ↔ ↑ CASI total score, CASI‐List‐generating, CASI‐attention, and composite score ● FC ↔ ↓ global cognitive function and attention
Chokesuwattanaskul et al. (2021)	17/–	25.2/60.6	NA/NA	PSG with extensive neuropsychological test, which included evaluation of executive functions using D‐KEFS tower test, design fluency test, and color‐word interference test, and evaluation of attention using WAIS‐IV Digit Span	3 T	3D T1‐w, T2‐FLAIR, and DTI	● Severe desaturation versus mild desaturation → ↓ cortical thickness in right inferior frontal and parietal gyri ● High ODI versus low ODI → ↓ cortical thickness in superior parietal gyrus ● Structural changes → central executive network ↔ executive function and attention ● Compensatory mechanism → ↑ activation or ↓ lateralization (→ preserved functions)
Yan et al. (2021)	68/21	27/46.3	23.9/3.5	PSG with standardized intelligence test, and NP tests assessing attention/executive function using Digit Span Test, Corsi Block Tapping Tests forward/backward, TMA/TMB, and Stroop Test	3 T and 8‐channel phased‐array coil	DSC	● OSA → ↓ global and regional CBF and CBV in default mode and attention networks (bilateral parietal and prefrontal cortices) ● Regional hypoperfusion ↔ intermittent hypoxia (parietal cortex) and sleep fragmentation (orbitofrontal cortex) ● Reference subjects → significant association in default mode and attention networks
Ramos et al. (2021)	16/–	29.9/52.6	NA/NA	PSG with cognitive function assessed using a customized computerized‐based cognitive assessment battery (NeuroTrax), which evaluated attention, executive function, verbal/visual memory, and speed of information processing via go‐no‐go response inhibition test, Stroop interference test, catch game test, staged information processing speed test, verbal/non‐verbal memory tests validated depression/anxiety scales were also used	3 T	3D T1‐w MPRAGE	● The ISI score and average oxygen levels ↔ brain volumes and cognition ● The ISI score → ↓ caudal anterior cingulate cortex and inferior parietal gyrus ● The ESS ↔ lateral ventricles ● Average oxygen saturation ↔ total cortical volume, lateral and medial orbitofrontal cortex, middle temporal cortex, and precuneus
Kong et al. (2021)	83/84	26.8/51.3	22.6/2.5	PSG with ESS used to assess sleepiness, and MoCA used to evaluate cognitive function, including executive functions, orientation, abstraction, attention, calculation, conceptual thinking, language, and memory	3 T and 8‐channel phased‐array head coil	rs‐fMRI	● OSA → abnormal FC of ventral, dorsal, and posterior insula ↔ cognitive esp. attention, emotional, and sensorimotor networks
Agarwal et al. (2022)	17(+SAD)/9(−SAD)	34.4/45.9	32.9/40.1	CPAP use > 6 h/day and a CNN model was used to classify MR images into +SAD and −SAD categories	NA	DTI	● CNN model ↑ accuracy (97.02%) in +SAD and −SAD classification ● CNN model ↑ participant‐level accuracy (99.11%) and image‐level accuracy (97.45%) at 90% probability threshold
Lee et al. (2022)	417(Persistent OSA)/458	25.6/14.3	23.7/1.6	PSG with a NP assessment battery, including SR and VR tests (immediate recall, delayed recall, and recognition), phonemic verbal fluency, categorical verbal fluency, digit symbol‐coding, TMA, and Stroop Test‐Word Reading and Color Reading	1.5 T	T1‐w and DTI	● OSA → ↓ cognition and WM integrity in 4 years ● Incident and persistent OSA → ↓ attention, visual processing, and visual memory ● ↓ Cognitive function ↔ changes in FA of WM areas
He et al. (2022)	18/18	NA/73.1	NA/2.5	PSG with neuropsychological assessments including MMSE, TMA/TMB, DST‐forward/backward, and RAVLT‐immediate recall/delayed recall/learning/forgetting	3 T	rs‐fMRI	● OSA patients → ↓ neuropsychological test performance than HCs ● Eight RSNs identified: DMN, DAN, VAN, and SN ● OSA patients → ↓ FC in bilateral PCC (DMN), right MFG (DAN), left STG (VAN), and ↑ FC in right SFG (SN)

**Abbreviation**: ACC, anterior cingulate cortex; AHI, apnea‐hypopnea index; aPFG, anterior prefrontal gyri; BDI, Beck depression inventory; BSC, best supportive care; CASI, cognitive abilities screening instrument; CBF, cerebral blood flow; CBV, cerebral blood volume; CC, corpus callosum; CDR, clinical dementia rating; Cho, choline; CNN, convolutional neural network; CPAP, continuous positive airway pressure; Cr, creatine; DAN, dorsal attention network; D‐KEFS, Delis–Kaplan Executive Function System; DMN, default mode network; DMS, delayed matching‐to‐sample; DST, digit span test; DTI, diffusion tensor imaging; EDS, excessive daytime sleepiness; ESS, Epworth Sleepiness Scale; FA, fractional anisotropy; IFG, inferior frontal gyri; ISI, insomnia severity index; LMT, Lagrange multiplier test; MD, mean diffusivity; MFG, middle frontal gyri; MMSE, mini mental state evaluation; MoCA, Montreal Cognitive Assessment; MSLT, multiple sleep latency test; MWT, maintenance of wakefulness test; NAA, *N*‐acetyl‐aspartate; NP, neuropsychological; ODI, oxygen desaturation index; PASAT, paced auditory serial addition test; PCC, posterior cingulate gyri; PSG, polysomnography; RAVL, Rey Auditory Verbal Learning; RD, radial diffusivity; ROI, region of interest; RSN, resting‐state networks; SAD, sustained attention deficit; −SAD, without sustained attention deficit; +SAD, with sustained attention deficit; SaO_2_, oxygen saturation of arterial blood; SF‐36, short form‐36 health survey; SFG, superior frontal gyrus; SN, salience network; SpO_2_, oxygen saturation; SR, story recall; STG, superior temporal gyri; TMA, trail making test A; TMB, trail making test B; TMT, trail making test; TPN, task positive network; VAN, ventral attention network; VBM, voxel‐based morphometry; VPSG, video‐polysomnography; VR, visual reproduction; WAIS‐IV, Wechsler Adult Intelligence Scale Fourth Edition; WMS‐IV, Wechsler Memory Scale Fourth Edition.

**FIGURE 2:**
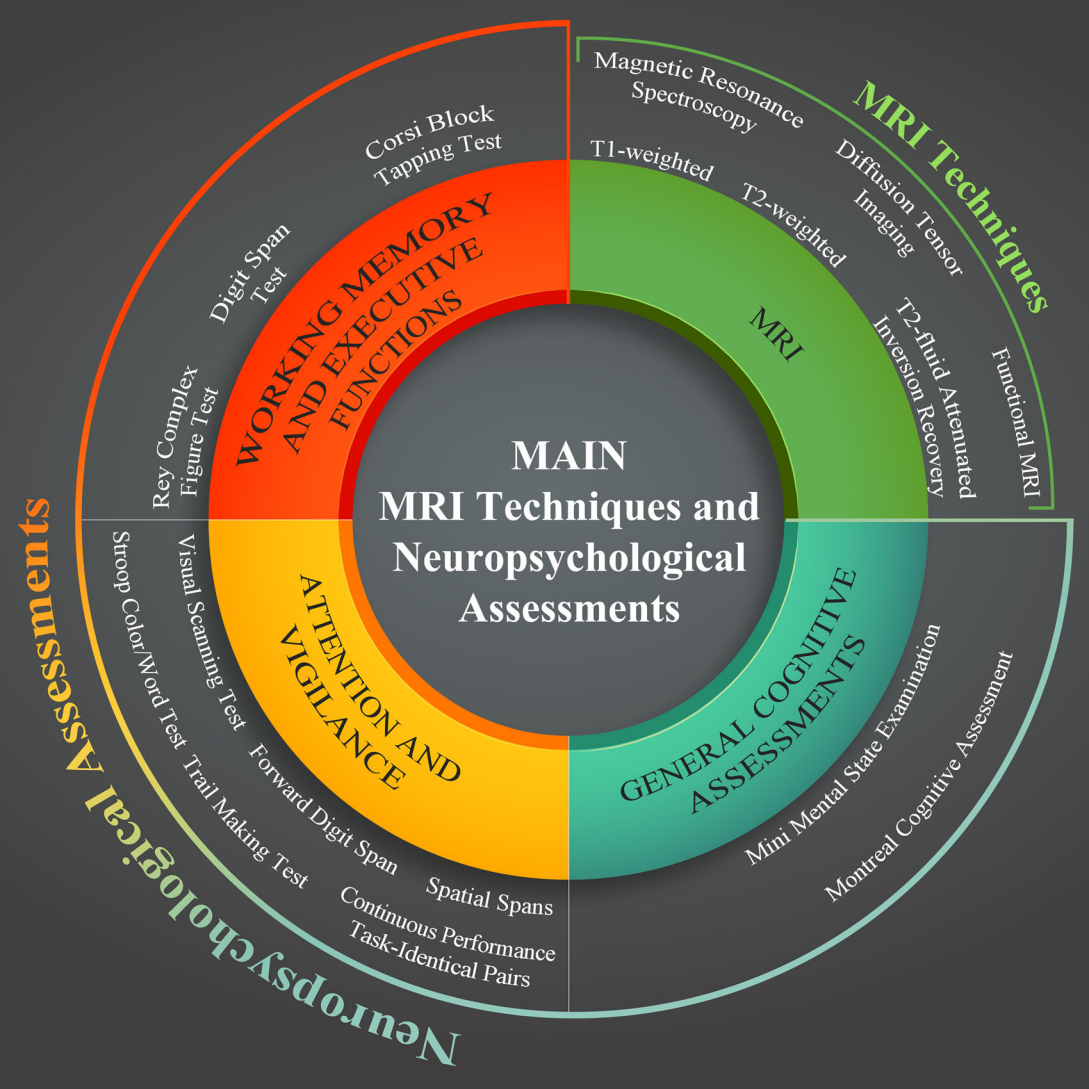
Main magnetic resonance imaging (MRI) techniques and neuropsychological assessments using the included studies.

Various MRI techniques and neuropsychological assessments were employed to investigate the relationship between OSA and attention deficits. These techniques included T1‐weighted imaging (T1‐w), T2‐weighted imaging (T2‐w), T2‐fluid attenuated inversion recovery (T2‐FLAIR), MRS, DTI, fMRI, resting‐state fMRI (rs‐fMRI), and others (Figure 2). Thirteen of the 20 studies employed structural MRI to examine brain alterations in OSA patients, including T1‐w, T2‐w, FLAIR, and DTI. Five of the 19 studies employed task‐based fMRI, or rs‐fMRI, to assess changes in brain function and FC in OSA patients. One study used MRS (Tonon et al., 2007), whereas another study employed the PWI technique (dynamic susceptibility contrast (DSC) method) (Yan et al., 2021).

The studies used a wide array of assessments to evaluate attention, vigilance, working memory, executive functions, and episodic memory. Tests, such as the Visual Scanning Test, Forward Digit Span, Spatial Spans, Stroop Color/Word Test, Trail Making Test, and Continuous Performance Task‐Identical Pairs, were used to measure attention and vigilance. Tests like the Digit Span Test, Corsi Block Tapping Test, and Rey Complex Figure Test were employed to evaluate working memory and executive functions. In some studies, the Mini Mental State Evaluation and Montreal Cognitive Assessment were used as general cognitive assessments (Figure 2).

OSA is associated with structural and functional brain alterations in various regions, especially in the frontal, parietal, temporal, and insular cortices, as well as the hippocampus, thalamus, basal ganglia, and cerebellum. These regions are involved in attention, memory, executive function, emotion, and sensorimotor processing. OSA is also associated with impaired cognitive performance in these domains, as well as global cognitive function, mood, and sleepiness. The cognitive impairments are correlated with the severity of OSA, as measured by polysomnography parameters, such as AHI, oxygen desaturation index (ODI), nadir oxygen saturation (SaO_2_), and arousal index.

**FIGURE 3:**
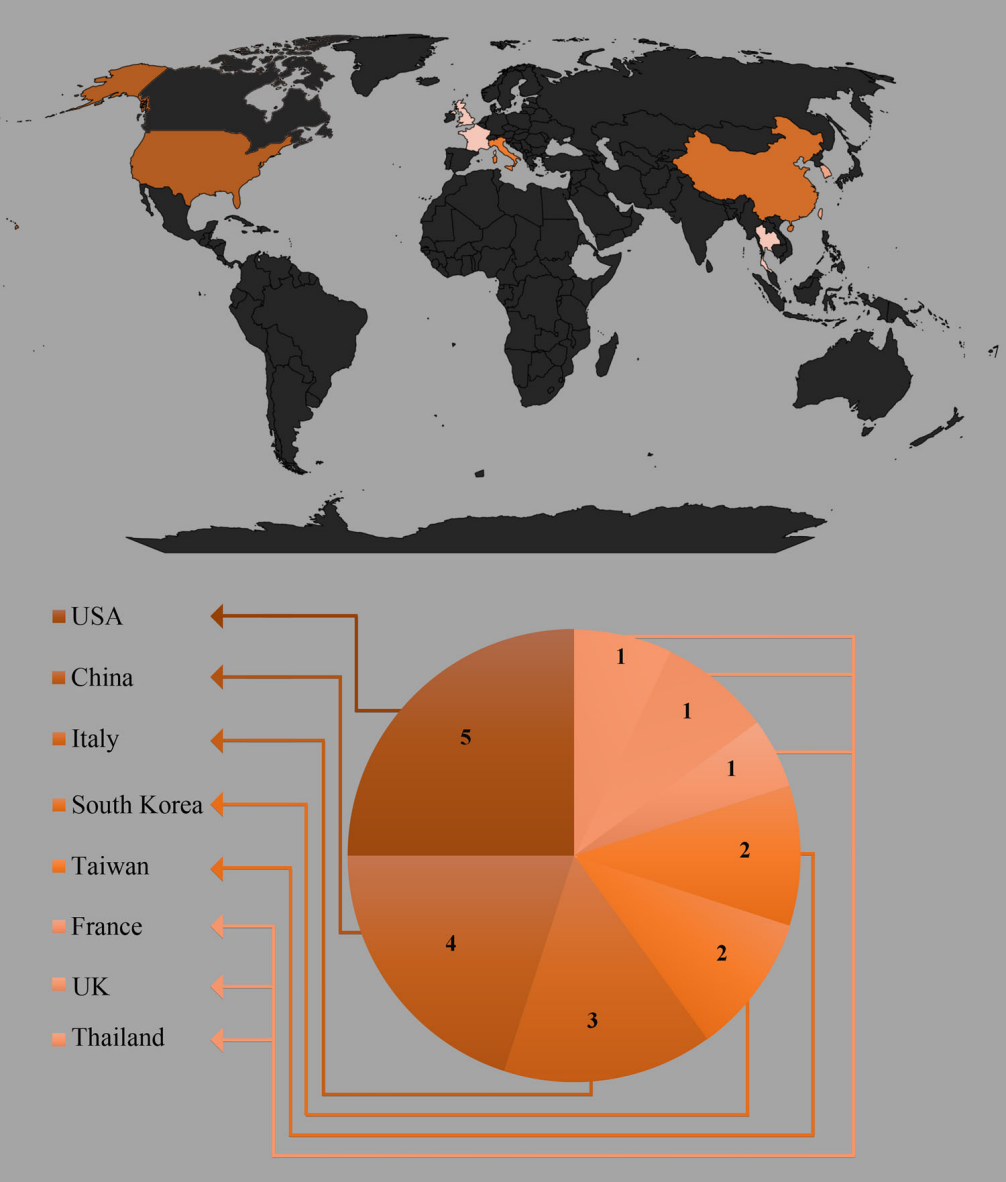
Geographic distribution of the countries and regions included in the systematic review of magnetic resonance imaging (MRI) biomarkers and neuropsychological assessments for obstructive sleep apnea (OSA) and attention deficits. The color gradient shows the proportion of articles from each country.

In our analysis of global population diversity and heterogeneity in OSA and attention research involving MRI and neuropsychological assessments, we discovered a geographical distribution pattern (Figure 3). Out of the 19 articles included in our study, the majority of the contributions came from the United States (*n* = 5), China (*n* = 4), and Italy (*n* = 3), collectively accounting for 63% of the articles. Furthermore, East and Southeast Asian countries, such as China, South Korea (*n* = 2), Taiwan (*n* = 2), and Thailand (*n* = 1), exhibited the highest participation rate across continents, with a 47% share (9 out of 19 articles).

Lastly, European countries, including Italy, France (*n* = 1), and the United Kingdom (*n* = 1), were responsible for 26% of the research (5 out of 19 articles). Notably, countries from the African continent, Oceania, Southwest Asia and the Middle East, Eastern Europe, and South America were not represented in the patient populations included in this study. Considering these findings, we recommend that future research efforts focus on increasing population heterogeneity by encouraging diagnostic interventions related to MRI and neuropsychological assessments in underrepresented regions. Such an approach will enhance the generalizability of subsequent review studies and provide a more comprehensive understanding of OSA's global impact. Additionally, conducting further investigations across diverse populations and countries will shed light on previously undiscovered aspects of the condition.

### 3.2 Summary of findings

#### 3.2.1 Structural MRI findings

GMV reduction has been found in the frontal, temporal, and parietooccipital cortices, the hippocampus, the basal ganglia, the thalamus, and the cerebellum, mostly in the right hemisphere (Canessa et al., 2011; Lin et al., 2016; Torelli et al., 2011; Yaouhi et al., 2009). A couple of studies (Chokesuwattanaskul et al., 2021; Joo et al., 2013) discovered dorsolateral prefrontal cortex atrophy or cortical thinning in the inferior parietal lobule and superior parietal gyrus. These alterations have been linked to attention deficits and other cognitive impairments. In some cases, treatment with CPAP has been shown to improve GMV and cognitive function (Canessa et al., 2011; Rosenzweig et al., 2016) although the extent of improvement can depend on treatment compliance (Castronovo et al., 2014).

DTI findings have revealed reduced fractional anisotropy (FA) and higher mean (MD) and radial diffusivity (RD) in various brain regions, such as the corpus callosum (CC), prefrontal cortex, and premotor and supplementary motor cortex (SMC), which are associated with impaired prospective memory and sustained attention in OSA patients (Zhang et al., 2019). In the study comparing OSA patients to HCs, studies have discovered abnormalities in the integrity of the WM or fiber damage in the anterior CC, anterior cingulate gyrus, and frontoparietal network (Castronovo et al., 2014; Ramos et al., 2021; Zhang et al., 2019). Specifically, DTI biomarkers indicated higher RD in the prefrontal cortex, as well as lower FA and higher MD and RD in the premotor and supplementary motor cortex. A noteworthy association was observed between reduced anterior CC integrity and impaired prospective memory and sustained attention in OSA patients. Additionally, OSA was found to negatively impact cognition and WM integrity over a 4‐year span (Lee et al., 2022). Both incident and persistent OSA were linked to decrements in attention, visual processing, and visual memory (Lee et al., 2022). These cognitive declines were associated with changes in FA of WM regions. Furthermore, reductions in cognition, mood, and sleepiness corresponded with decreased WM fiber integrity (FA and MD) across multiple brain areas in pretreatment OSA patients (Castronovo et al., 2014). After 3 months of CPAP intervention, only limited changes in WM were observed. In contrast, after 12 months of CPAP treatment, compliant patients exhibited improvements in WM integrity. These WM changes posttreatment were positively correlated with enhanced attention and executive functioning, emphasizing the importance of CPAP compliance for achieving favorable outcomes (Canessa et al., 2011). Finally, Agarwal et al. (2022) employed a convolutional neural network model to accurately categorize MR images into sleep apnea duration (+SAD) and non‐sleep apnea duration (−SAD) categories (Agarwal et al., 2022).

#### 3.2.2 Functional and metabolic MRI findings

Some studies revealed lower or altered FC in many resting‐state networks (RSNs), including the default mode network (DMN) (Chang et al., 2020; He et al., 2022; Prilipko et al., 2012), dorsal attention network (DAN) (He et al., 2022), ventral attention network (VAN) (He et al., 2022), and salience network (SN) (He et al., 2022). The cingulate cortex, prefrontal cortex, middle frontal gyrus (MFG), inferior frontal gyrus (IFG), and middle and superior temporal regions are common among these networks. Some studies also discovered that when cognitive activities, such as working memory, attention, and mismatch processing, were performed, the ACC, MFG, and IFG were less active, in contrast right anterior prefrontal gyrus increased activity (He et al., 2022; Zhang et al., 2011). In another fMRI study, Prilipko et al. (2012), found that OSA patients had less deactivation in the DMN and TPN. Treatment with CPAP has been shown to modulate cerebral activation and deactivation patterns, leading to improved behavioral performance (Prilipko et al., 2012).

In OSA patients, abnormal FCs of the ventral anterior insula, dorsal anterior insula, and posterior insula were seen in several brain regions (Kong et al., 2021). Some studies have reported associations between FC and OSA severity indices, such as the AHI (Chang et al., 2020), ODI (Chang et al., 2020; Chokesuwattanaskul et al., 2021), and SaO_2_ (Chang et al., 2020). OSA patients had substantially lower FC in the attention‐related networks, such as the bilateral posterior cingulate gyri of the DMN, the right MFG of the DAN, and the left superior temporal gyrus (STG) of the VAN (He et al., 2022). OSA patients also had higher FC in the right superior frontal gyrus of the SN (He et al., 2022).

In regards to other perfusion and metabolic techniques, one study using DSC has shown reduced global and regional CBF and cerebral blood volume (CBV) in a variety of cortical and deep regions associated with the DMN and attention networks, primarily in bilateral parietal and prefrontal cortices (Yan et al., 2021). These reductions have been linked to intermittent hypoxia and sleep fragmentation (Yan et al., 2021). Another study used MRS to measure cortical *N*‐acetylaspartate (NAA), creatine, and choline (Cho) concentrations and discovered that baseline cortical NAA concentration was significantly lower in OSA patients. After 6 months of CPAP treatment, no changes were detected (Tonon et al., 2007). Finally, one multimodal neuroimaging found that OSA patients had a decrease in brain metabolism that was more restricted than GM density changes and involved regions, such as the precuneus, the cingulate gyrus, and the parietooccipital cortex, as well as the prefrontal cortex (Yaouhi et al., 2009).

#### 3.2.3 Neuropsychological findings

When compared to HCs or baseline measures, most studies found that OSA patients had complications with attention, especially sustained attention (Canessa et al., 2011; Castronovo et al., 2014; Joo et al., 2013; Prilipko et al., 2012; Torelli et al., 2011; Zhang et al., 2011). Other some studies discovered that CPAP therapy resulted in changes in the structure or function of brain regions involved in attention (Canessa et al., 2011; Castronovo et al., 2014; Lin et al., 2016; Prilipko et al., 2012; Rosenzweig et al., 2016). A few studies have found correlations between attention performance and polysomnography parameters, such as AHI, ODI, nadir SaO_2_, arousal index, and insomnia severity index, or brain measures, such as GMV, WM integrity, FC, and CBF (Castronovo et al., 2014; Chang et al., 2020; Joo et al., 2013; Ramos et al., 2021; Rosenzweig et al., 2016; Tonon et al., 2007; Zhang et al., 2011; Zhang et al., 2019). Treatment with CPAP has been shown to improve cognitive function in various domains, such as attention (Canessa et al., 2011; Prilipko et al., 2012; Rosenzweig et al., 2016). However, not all studies have reported improvements in cognitive performance following treatment (Tonon et al., 2007). Moreover, the extent of cognitive recovery may be influenced by some factor such as treatment compliance (Castronovo et al., 2014).

## 4 DISCUSSION

**FIGURE 4:**
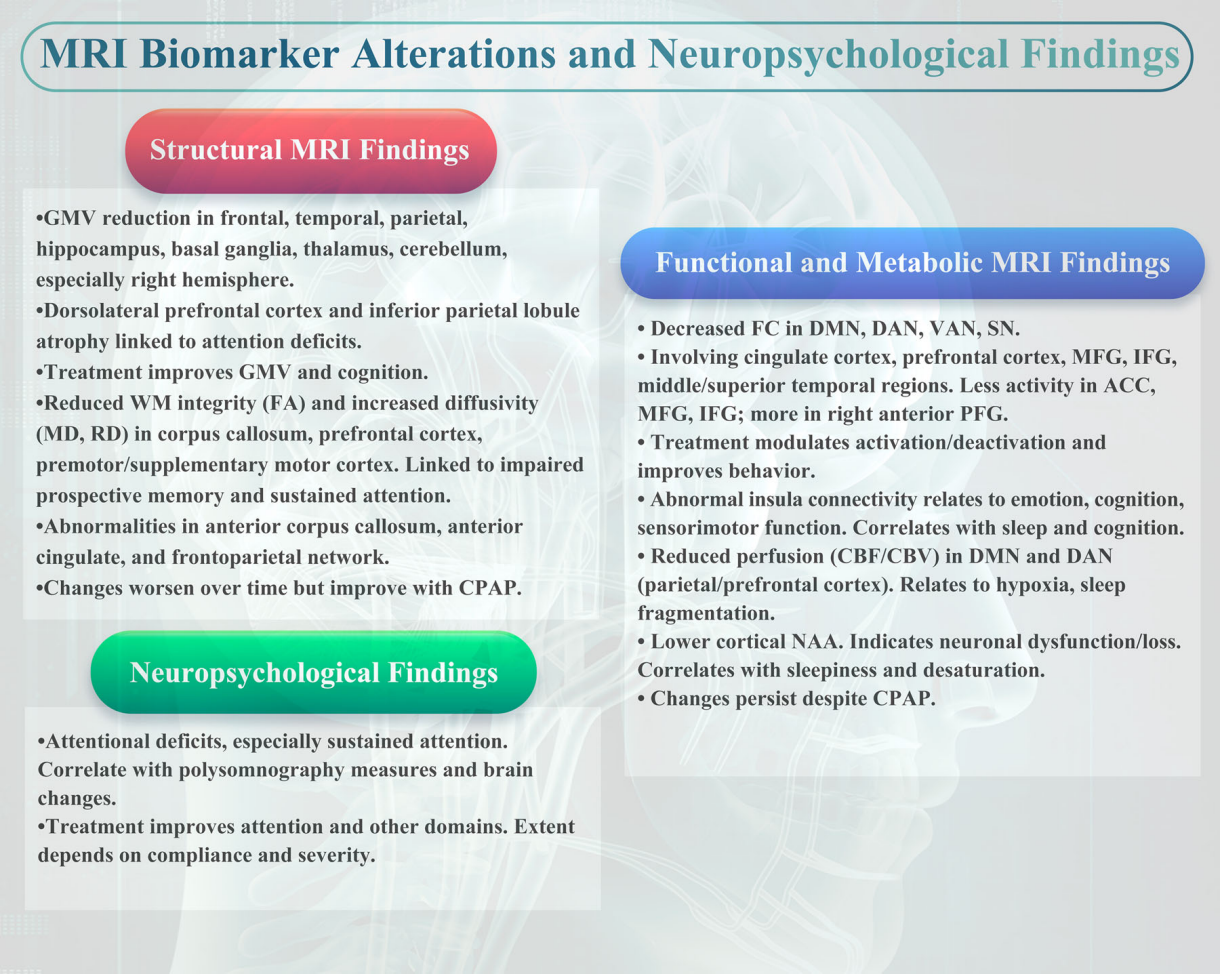
Main magnetic resonance imaging (MRI) biomarker alterations and neuropsychological assessments in obstructive sleep apnea (OSA) patients.

The present study aimed to review the MRI and neuropsychological characteristics and findings in attention deficits of OSA. This review provides an updated and comprehensive overview of the recent advances in MRI techniques and neuropsychological assessments that have been applied to OSA research. According to the systematic review, OSA has significant effects on brain structure and function, which could lead to attention deficits (Figure 4).

One of the main findings of this review is that OSA is associated with structural alterations in several brain regions that are involved in attention processing. The majority of the studies that used structural MRI reported reduced GMV or cortical thickness in multiple brain regions in OSA patients, especially in the frontal (Canessa et al., 2011; Chokesuwattanaskul et al., 2021; Joo et al., 2013; Yaouhi et al., 2009), temporal, and parietal lobes, as well as the hippocampus (Canessa et al., 2011; Lin et al., 2016; Yaouhi et al., 2009), thalamus (Yaouhi et al., 2009), caudate, and insula (Canessa et al., 2011; Chokesuwattanaskul et al., 2021; Joo et al., 2013; Lin et al., 2016; Prilipko et al., 2012; Torelli et al., 2011). These structural deficits were associated with cognitive impairments such as attentional disorder (Canessa et al., 2011; Joo et al., 2013; Lin et al., 2016; Torelli et al., 2011), which are commonly impaired in OSA patients. The GMV or cortical thickness reductions were also associated with OSA severity indices, such as AHI, ODI, SaO_2_, arousal index, and Epworth sleepiness scale. Some studies also found higher GMV or cortical thickness in some regions, such as the cerebellum, precuneus, ACC, and PFG, which can reflect compensatory mechanisms or recovery from edema after treatment (Canessa et al., 2011; Lin et al., 2016). Canessa et al. (2011) showed after treatment, significant improvements in attention were observed, which paralleled GMV increases in hippocampal and frontal structures (Canessa et al., 2011). Treatment with CPAP was shown to partially reverse some of the GMV or cortical thickness changes and improve cognitive function in some studies (Canessa et al., 2011; Castronovo et al., 2014). Cognitive (such as attentional disorder) and structural deficits in OSA can be secondary to sleep deprivation and repetitive nocturnal intermittent hypoxemia (Yan et al., 2021). These findings are in line with Lin et al. (2016), who showed after treatment, improvements in attention and executive‐functioning paralleled GMV increases in hippocampal and frontal structures (Lin et al., 2016). Furthermore, preoperatively, patients presented with worse cognitive function,and worse polysomnography scores associated with higher insular GMV (Lin et al., 2016).

The studies that used DTI reported reduced WM integrity or fiber density in several brain areas in OSA patients, mainly in the CC, prefrontal cortex, premotor cortex, SMC, and cingulum (Castronovo et al., 2014; Chokesuwattanaskul et al., 2021; Lee et al., 2022; Zhang et al., 2019). These regions are important for interhemispheric communication, motor control, and attention regulation (Boussaoud, 2001; Simon et al., 2002). The WM integrity or fiber density reductions were also correlated with OSA severity indices and cognitive performance measures, such as prospective memory and sustained attention (Koo et al., 2023; Mullins et al., 2020). Furthermore, CPAP treatment was found to improve WM integrity or fiber density in some regions and enhance attention and executive function in some studies that were approved by previous studies (Lajoie et al., 2020; Salsone et al., 2021).

Attention is supported by multiple brain networks that interact dynamically depending on the task demands and environmental context (Denkova et al., 2019; Kao et al., 2020; Parks & Madden, 2013; Zhou et al., 2023). These networks include the DAN, which mediates top–down control of attention; the VAN, which mediates bottom–up detection of salient stimuli; the DMN, which mediates internally oriented attention; and the SN (Yousaf et al., 2017), which mediates switching between external and internal attention (Corbetta & Shulman, 2002; Dragomir & Omurtag, 2020; Menon & Uddin, 2010; Su et al., 2021). These networks are responsible for maintaining internal mental states, orienting attention to external stimuli, detecting salient events, and switching between other networks (Lindsay, 2020). The abnormal RSNs were associated with worse performance on neuropsychological tests (Benito‐León et al., 2015; He et al., 2022). Besides, Kong et al. (2021) reported abnormal insular connectivity related to emotion, cognition, and sensorimotor function, which correlated with sleep and cognitive variables. These findings suggest that OSA imapirs attention and other higher order functions by disrupting FC within and between RSNs (Ayalon et al., 2009; Zhang et al., 2013). The FC or activation changes were also related to OSA severity indices and cognitive performance measures, such as global cognitive function, attention span, working memory, response time, and error rate (Ayalon et al., 2009; Park et al., 2021).

CPAP treatment was shown to modulate FC or activation in some regions and improve behavioral performance in some studies, but not in others. These inconsistent results may be due to different fMRI paradigms, analysis methods, or baseline characteristics (Khazaie et al., 2017; Li et al., 2022; Long et al., 2023; Prilipko et al., 2012; Prilipko et al., 2014; Sun et al., 2023). Moreover, OSA triggers compensatory or adaptive responses in the brain, such as increased activation or deactivation in specific regions or networks, increased connectivity or plasticity in others, or higher brain volume or metabolism in some areas. These responses reflect an attempt to maintain cognitive function or cope with OSA's adverse effects (Chou et al., 2019; Tahmasian et al., 2016).

The DSC and MRS studies provide insight into the neural mechanisms underlying the attention deficits in OSA (Tonon et al., 2007; Wallin et al., 2018; Yan et al., 2021). Reduced CBF and CBV were observed in the DMN and DAN, including the bilateral parietal and prefrontal cortices. These regions are critical for sustained attention and attention allocation (Chang et al., 2020; Orosz et al., 2012; Schneider et al., 2022; Zhang et al., 2018). The hypoperfusion in these attention networks could contribute to the impaired attention and vigilance commonly reported in OSA patients (Faria et al., 2021; Legault et al., 2021). The reduced CBF and CBV were also associated with OSA severity measures like lower minimum SpO_2_ and sleep fragmentation, suggesting that the neural abnormalities are related to the physiological impacts of OSA (L'Heureux et al., 2021; Macey, 2015). Specifically, chronic intermittent hypoxia may lead to perfusion deficits in the parietal cortex, whereas sleep fragmentation could affect the orbitofrontal cortex (Baril et al., 2015; Daulatzai, 2012; Innes et al., 2015; Rosenzweig et al., 2016).

In addition, OSA patients exhibited lower NAA concentrations in the cortical regions at baseline compared to HCs (Tonon et al., 2007). NAA is a marker of neuronal integrity and function (Chen et al., 2022; Kalra, 2019; Moffett et al., 2007). The lower NAA levels indicate neuronal dysfunction or loss, which could underlie the attention and cognitive impairments (Benarroch, 2008; Schuff et al., 2006). The reduced NAA concentrations were also correlated with worse sleepiness and oxygen desaturation in OSA, highlighting their clinical relevance (Kainulainen et al., 2019; Meliante et al., 2023; Wali et al., 2020). Despite significant improvements in EDS, sleep fragmentation, and oxygen saturation following CPAP treatment, the cortical perfusion, NAA concentrations, and cognitive functions did not normalize after 6 months of CPAP therapy in some studies. The persistent neural abnormalities may explain the residual attention and cognitive deficits even after CPAP treatment in some OSA patients (Liu et al., 2020; Xiong et al., 2017).

To sum up, the structural, functional, and metabolic neuroimaging evidence converges to indicate that OSA affects multiple brain networks subserving attention, particularly front‐parietal systems. The diffuse patterns of GM atrophy, WM disruption, hypoperfusion, and altered connectivity suggest OSA has a systemic impact on brain structure and function. Notably, the attention networks most strongly implicated—the DAN, VAN, and SN—play key roles in top–down attentional control, bottom–up stimulus detection, and switching between internal versus external attention. This aligns with the attentional deficits frequently reported in OSA. The involvement of both dorsal and ventral frontoparietal circuits points to OSA disrupting both goal‐directed and stimulus‐driven attention. Another key finding is that while some studies show recovery in brain structure/function and cognition following CPAP treatment, others show persistent deficits. This suggests individual variability in the reversibility of brain changes, which may depend on factors like OSA severity, neural compensatory capacity, and treatment compliance. Clinically, a subset of patients may require more aggressive management to fully reverse attention deficits.

## 5 LIMITATIONS AND RECOMMENDATIONS

It is important to take into account certain limitations. The utilization of various MRI techniques and neuropsychological tests in different studies can result in differences in the ability to detect changes in the brain and cognitive functions. To mitigate these inconsistencies, it would be helpful to standardize the protocols for structural, functional, and metabolic MRI, as well as cognitive assessment batteries that are specifically designed for patients with OSA. Larger sample sizes, especially for follow‐up studies, are needed to confirm preliminary findings on the effects of OSA treatment. Future research should control for these potential confounds, which could also influence brain structure, function, and cognition. Another limitation is the paucity of longitudinal studies investigating structural brain changes and cognitive decline over time in OSA. Most studies employed cross‐sectional rather than longitudinal designs. Further high‐quality longitudinal research is critically needed to delineate progressive brain and cognitive changes in OSA and tease apart the effects of the disease process from potential confounds. Long‐term follow‐up of OSA patients and the employment of machine learning techniques are vital to facilitate the detection of complex patterns in multimodal imaging and cognitive decline with early diagnosis and treatment.

## 6 CONCLUSIONS

This review provides an updated and comprehensive summary of the MRI biomarkers and neuropsychological assessments of attention deficits in patients with OSA. We found that OSA is associated with structural and functional brain alterations in multiple regions and networks that are involved in attention processing, such as the frontal, temporal, and parietal lobes, the hippocampus, the thalamus, the insula, the CC, the ACC, the middle and inferior frontal gyri, the posterior cingulate cortex, and the STG. These brain changes are also correlated with OSA severity indices and cognitive performance measures, such as global cognitive function, attention span, working memory, response time, and error rate. Treatment with CPAP can partially reverse some of the brain changes and improve cognitive function in some domains and in some studies, but not in others. The inconsistent results may be due to methodological differences or confounding factors.

## AUTHOR CONTRIBUTIONS

**Sadegh Ghaderi**: Conceptualization; data curation; investigation; methodology; project administration; supervision; validation; visualization; writing—original draft; writing—review and editing. **Sana Mohammadi**: Data curation; investigation; methodology; validation; visualization; writing—original draft; writing—review and editing. **Mahdi Mohammadi**: Data curation; investigation

## FUNDING INFORMATION

No specific funding was received for this work.

## CONFLICT OF INTEREST STATEMENT

No conflicts of interest were identified by the authors.

### PEER REVIEW

The peer review history for this article is available at https://publons.com/publon/10.1002/brb3.3262

## Data Availability

This article contains all of the data produced or analyzed during this investigation. Any further inquiries should be forwarded to the corresponding author.
